# Advanced hitchhiking nanomaterials for biomedical applications

**DOI:** 10.7150/thno.88002

**Published:** 2023-08-28

**Authors:** Ying Wang, Shao-Kai Sun, Yang Liu, Zhanzhan Zhang

**Affiliations:** 1 School of Medical Imaging, Tianjin Medical University, Tianjin 300203, China.; 2 Tianjin Key Laboratory of Retinal Functions and Diseases, Tianjin Branch of National Clinical Research Center for Ocular Disease, Eye Institute and School of Optometry, Tianjin Medical University Eye Hospital, Tianjin 300384, China.; 3 Key Laboratory of Functional Polymer Materials of Ministry of Education, College of Chemistry, Nankai University, Tianjin 300071, China.

**Keywords:** hitchhiking, nanomaterials, circulatory cells, navigation, targeted delivery

## Abstract

Hitchhiking, a recently developed bio-inspired cargo delivery system, has been harnessed for diverse applications. By leveraging the interactions between nanoparticles and circulatory cells or proteins, hitchhiking enables efficient navigation through the vasculature while evading immune system clearance. Moreover, it allows for targeted delivery of nutrients to tissues, surveillance of the immune system, and pathogen elimination. Various synthetic nanomaterials have been developed to facilitate hitchhiking with circulatory cells or proteins. By combining the advantages of synthetic nanomaterials and circulatory cells or proteins, hitchhiking nanomaterials demonstrate several advantages over conventional vectors, including enhanced circulatory stability and optimized therapeutic efficacy. This review provides an overview of general strategies for hitchhiking, choices of cells and proteins, and recent advances of hitchhiking nanomaterials for biomedical applications.

## Introduction

With the rapid development of nanotechnology, drug delivery systems (DDS) based on engineered nanoparticles have demonstrated remarkable efficacy in various biomedical applications 1-3. Nanomaterials employed in drug delivery can be derived from diverse sources, offering versatility in their composition. In addition, their physical properties can be readily customized by manipulating factors such as size, shape, composition, surface characteristics, structure, and morphology 4-6. More importantly, nanomedicine offers several notable advantages over free drugs, including improved stability and solubility, enhanced therapeutic efficacy, and reduced toxic side effects 7-9. Capitalizing on these distinctive attributes, several types of nanomedicines have received approval from the Food and Drug Administration (FDA) for cancer treatment. Notable examples include Doxil® for Kaposi sarcoma and Lipolatin for non-small cell lung cancer 10, 11. These nanomedicines have significantly enhanced the clinical benefits of anticancer drugs, leading to a considerable extension in the survival of cancer patients. However, traditional nanomedicines still face several challenges, such as high systemic toxicity, low response rates, and limited durability, which hinder their widespread adoption in clinical settings 12. Therefore, addressing the means to further enhance the effectiveness and safety of nanomedicine becomes a crucial matter for future advancements in nanomaterials.

Cell-based therapies have a long-standing history, with the first recorded human blood transfusion dating back to the mid-17th century 13. In the era of modern medicine, significant progress has been achieved in harnessing the inherent capabilities of cells for targeted therapeutic interventions. These advancements encompass diverse applications, such as transfusions of specific blood components (such as platelets, plasma, white blood cells (WBC), or red blood cells (RBC)) 14, 15, mesenchymal stem cells for tissue regeneration 16-18, and immunotherapies for cancer treatment 19-21. Remarkably, the circulatory cells and proteins in the bloodstream, including RBC, macrophages, monocytes, lymphocytes, and serum albumin (SA), serve as nature's own “delivery vehicles”, has evolved to carry out delivery functions within living organisms efficiently 22-25. These unique properties make circulating cells/proteins promising candidates for the development of therapeutics that can overcome the limitations often associated with traditional nanomedicines.

Hitchhiking, in the context of cargo delivery, refers to using external carriers to transport cargo to its desired destination 26. Some organisms have evolved hitchhiking behavior in nature, where smaller organisms attach to larger ones and get transported to new locations 27, 28. Drawing Inspiration from this natural phenomenon, hitchhiking has recently been adapted as a bio-inspired cargo delivery system for various applications 29. The versatility of nanoparticles allows for their interaction with circulatory cells or proteins, thereby facilitating hitchhiking 3. In this system, circulatory cells or proteins play a pivotal role as innate “moving gears” that facilitate the transport of hitchhiking nanoparticles throughout the body, until reaching their desired target site 29, 30. Hitchhiking nanoparticles leverage the natural ability of circulatory cells or proteins to (i) navigate the vasculature while evading immune system clearance and (ii) perform specific tasks such as delivering nutrients to tissues, eliminating pathogens, and surveilling the immune system 22, 31-38. Additionally, hitchhiking nanoparticles can be used to genetically engineer and label specific cell types, such as macrophages, T cells, and B cells 37, 39, 40. This allows further customization and manipulation of these cells, enhancing their capabilities for targeted drug delivery and other therapeutic applications.

**Figure 1:**
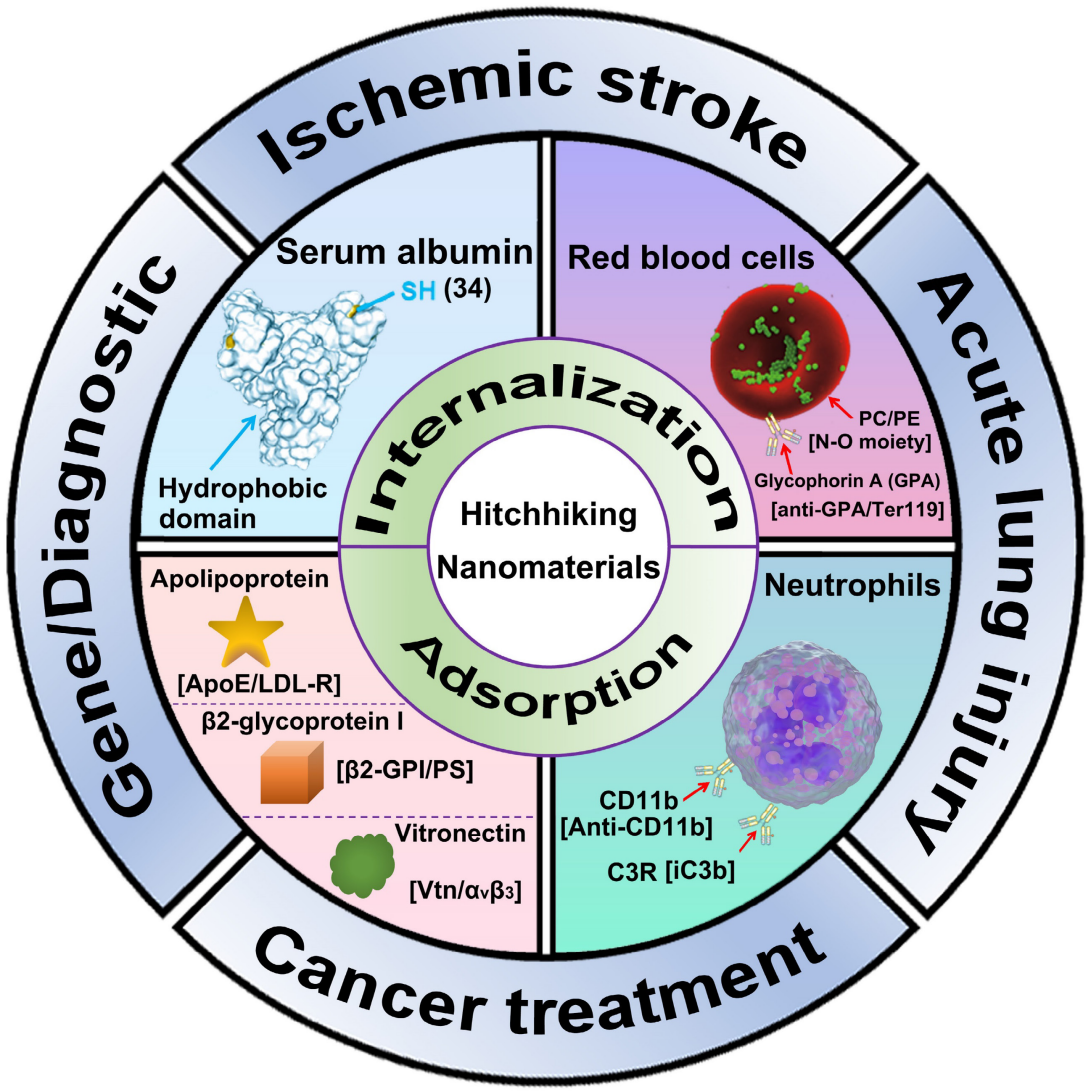
Schematic illustration of Advanced hitchhiking nanomaterials for biomedical applications. Advanced hitchhiking nanomaterials employ internalization-mediated or adsorption-medicated strategy to hitchhike circulatory cells and proteins for various biomedical applications, including ischemic stroke, acute lung injury, cancer treatment, gene therapy, and diagnostic imaging. Adapted with permission from 121, copyright 2023 American Chemical Society; 31, copyright 2018 Springer Nature.

This review introduces the general strategies for nanoparticle to hitchhiking circulatory cells or proteins, including internalization-mediated hitchhiking and adsorption-medicated hitchhiking (**Figure 1**). Next, the cell and protein choices for cellular hitchhiking (RBC, neutrophils, and SA) are demonstrated. Finally, we summarize the recent advances of hitchhiking nanomaterials in biomedical applications, including ischemic stroke, acute lung injury, cancer treatment, gene therapy, and diagnostic imaging.

## General strategies for hitchhiking

Circulatory cells are biological entities composed of biomolecules including polysaccharides, lipids, and proteins that provide a variety of functional groups and surface properties 41, 42. These characteristics allow for the utilization of various techniques to hitchhike these cells. The delivery of nanomaterials for cell-specific targeting represents a crucial step in achieving efficient cell hitchhiking. However, successfully and effectively implementing this strategy requires addressing several challenges. Notably, two major hurdles are associated with the stability and half-life of nanomaterials. One promising approach to improve nanoparticle storage and circulation stability is by encapsulating them within protective coatings or matrices, such as PEG and zwitterionic molecules 43, 44. These protective layers shield the nanomaterials from potential degradation and immune recognition, enhancing their overall stability during transport. Additionally, the design of intelligent release mechanisms offers a solution to ensure controlled and targeted payload release. These mechanisms can be engineered to respond to specific triggers, such as changes in pH or enzymatic activity, allowing for precise release of the payload at the desired target site 8, 45, 46. With these strategies, the stability of the nanomaterials can be further augmented, leading to an efficient and successful cell-specific targeting approach. In this section, we will briefly describe two major strategies for cell hitchhiking: (1) internalization-mediated hitchhiking and (2) adsorption-mediated hitchhiking. Furthermore, we will discuss the advantages and disadvantages of these two hitchhiking strategies.

### Internalization-mediated hitchhiking

One promising strategy for hitchhiking circulatory cells involves leveraging the natural ability of specific cells to phagocytose foreign nanomaterials, commonly referred to as the “Trojan Horse” method 47-49. In this strategy, the phagocytic capacity of cells is crucial for successful hitchhiking. Typically, this is achieved by incubating nanoparticles with cells capable of phagocytosis in a culture medium, allowing the nanoparticles to adsorption on cells' surface and subsequently cellular internalization. Using this strategy, various nanomaterials, including liposomes, polymeric nanoparticles, nanozymes, and metal-based nanomaterials, have been successfully incorporated into neutrophils and macrophages 48, 50-52. Importantly, this method preserves the integrity of the cell membrane and avoids the off-target effect of the nanomaterials *in vivo*. However, it is important to recognize that the origins of the “Trojan Horse” method lie in targeted drug delivery approaches that are used to kill cells, especially in the context of cancer nanomedicine 53. The premature release of loaded drugs during hitchhiking may disrupt the integrity of the cell membrane, thus impairing the migration, homing, and other functions of hitchhiked cells/proteins, resulting in unpredictable therapeutic efficacy. Therefore, optimize the release behaviors of loaded drugs to ensure the precisely controlled release of the drug only in the target is a prerequisite for this internalization-mediated hitchhiking. Besides, despite the high efficiency and specificity of this *in vitro* culture method, common issues associated with complex steps, such as cell extraction and purification, limit the generalizability of this strategy 54, 55. To overcome these limitations, direct *in vivo* hitchhiking strategies have been developed, primarily by utilizing the scavenging capability of macrophages and monocytes. For example, Wang *et al.* discovered that activated neutrophils could specifically endocytose denatured BSA nanoparticles via Fc*γ* receptor (Fc*γ*R) III *in vivo* 56. Building on this finding, they subsequently developed a series of denatured BSA-based nanomaterials for *in vivo* neutrophil hitchhiking. Similarly, Wang *et al.* demonstrated that a specific lipid called DOCP could rapidly accumulate complement fragment iC3b upon intravenous injection, triggering neutrophil hitchhiking through C3 receptor-mediated phagocytosis 57.

In addition to exploiting the scavenging capability of circulatory cells, ligand-receptor interaction is another way for nanomaterials to hitchhike circulatory cells. The ligand-receptor interaction refers to highly selective and complementary interactions between a ligand molecule and its corresponding receptor protein within a living organism 58, 59. Ligands are signaling molecules that can bind to specific receptors, usually present on the surface of cells, to trigger a cellular response 60, 61. This interaction is analogous to a lock and key mechanism, where only a specific ligand (the key) can fit into its corresponding receptor (the lock) to activate a particular biological pathway or process. This specificity prevents unwanted crosstalk between different signaling pathways and helps in achieving accurate cellular responses. For example, Stephan *et al.* presented an anti-CD3e f(ab')2-decorated polymeric nanoparticle and successfully hitchhiked and programmed leukemia-specific T cells through the CD3/anti-CD3 interaction 62. Similarly, Stavrou *et al.* reported an anti-Ly6G-intergrated liposome and effectively hitchhikes activated neutrophils through the Ly6G/anti-Ly6G interaction 63. This ligand-receptor attachment strategy offers several advantages, including reproducibility, reliability, specificity, and the potential for translation into *in vivo* hitchhiking when specific ligand-receptor pairs are involved. Furthermore, this approach allows for designing a platform technology that can attach to various cell types by modifying the attachment ligand on the nanoparticle surface, making it ideal for scalable particle technology. These innovative approaches offer promising alternatives for hitchhiking circulatory cells *in vivo*, circumventing the complexities associated with *in vitro* methods. However, it is worth noting that this strategy is easier to implement in cell types capable of phagocytosis, such as monocytes and macrophages. Furthermore, biodegradable nanoparticles may undergo intracellular degradation and release drugs after phagocytosis, thereby affecting the migratory function of the cells 64-66.

### Adsorption-mediated hitchhiking

Adsorption-mediated hitchhiking mainly occurs in circulatory cells that are non-endocytic or poorly endocytic, such as T cells, B cells, and RBCs. According to the different interaction modes between nanomaterials and cells, adsorption-mediated hitchhiking can be roughly divided into two categories: (1) non-specific adsorption driven by electrostatic interaction, van Der Waals forces, and hydrogen bonding; and (2) site-specific adsorption driven by ligand-receptor interactions. It is important to note that when nanoparticles are tethered on the cell surface, it becomes paramount to ensure stability and controlled dissociation from the cells. As circulatory cells transport nanoparticles through the bloodstream, they encounter significant shear forces 67, 68. Proper stability of the nanoparticle-cell complex is crucial for preventing the premature release of nanoparticles during the hitchhiking process. Therefore, an ideal nanoparticle that relies on adsorption for *in vivo* hitchhiking should consider the following aspects when designing: 1) The binding affinity between nanoparticles and cells should be strong enough to withstand shear forces during blood circulation, and 2) controlled release from hitchhiked cells upon reaching targets for optimized therapeutic efficacy and minimized toxic effect. However, the current hitchhiking nanomaterials only consider enhancing the interaction with the cells while ignoring the controlled release. This may be because the side effects caused by the destruction of a small number of hitchhiking cells are largely ignored. In addition, long-term interactions with the cell surface may promote the cellular internalization of nanoparticles, which may affect the original function of nanoparticles. Therefore, resistance to cellular endocytosis should also be an important property of these nanomaterials.

#### Non-specific adsorption

One of the simplest methods for attaching nanoparticles to cells is passive adsorption on the cell surface. The surface charge, shape, and size of nanoparticles are the three important parameters that affecting their non-specific adsorption onto cells. (1) Surface charge. The mammalian cells usually possess a negative-charged surface due to negatively charged groups such as sialic acid, carboxylate, and phosphate on biomolecules 69. This inherent characteristic of cells offers a straightforward approach to attaching cationic nanoparticles to their surface through electrostatic interactions. For example, Manshian *et al.* discovered that polyethyleneimine-coated poly (lactic-co-glycolic acid) nanoparticles (PEI-PLGA, 17.3 ± 0.4 mV) demonstrated a twofold higher binding efficacy to RBCs than that of plain PLGA NPs (-25.9 ± 0.2 mV) 70. Similarly, Nikitin *et al.* presented that chitosan-coated nanoparticles (42.1 ± 8.8 mV) strongly bind to RBCs even after multiple washes 71. However, it is essential to note that while these positively charged nanoparticles exhibit strong adsorption to cell membranes, excessive positive charges may lead to cellular internalization, cell membrane dissolution and potential toxicity 72, 73. (2) Particle size. Nanoparticle size is a crucial factor influencing cellular adsorption efficiency. Smaller nanoparticles have been demonstrated to exhibit higher cellular uptake efficiency 74, 75. For instance, Liu *et al.* conducted a study wherein nano-sized aggregates with diameters of 132.6 nm (CA-5) displayed slower uptake kinetics compared to smaller-sized particles such as CA-1 (46.9 nm) and CA-3 (37.59 nm) 76. Consequently, nanoparticles with suitable sizes, generally larger than 100 nm, are expected to yield benefits in adsorption on the cell surface. (3) Particle shape. Nanoparticles of different shapes possess distinct surface areas relative to their volumes. Those with larger surface areas tend to establish stronger interactions with cell surfaces due to increased contact points 77, 78. However, such strong interactions might also lead to enhanced cellular internalization. For instance, cubes exhibit the lowest cellular uptake efficacy among various shapes, followed by cylinders, spheres, and rods. In light of these findings, achieving a delicate balance between enhancing cell interactions and avoiding excessive endocytosis becomes crucial for successfully applying non-specific adsorption-mediated hitchhiking. In addition, van Der Waals forces and hydrogen bonds also contribute greatly to non-specific adsorption 79, 80. For example, Shen *et al.* found that *N*-oxide moiety can reversibly bind to RBCs membranes through interaction with the hydrophilic groups of phosphatidylethanolamines (PE) and phosphatidylcholine (PC) 81. In addition, this zwitterion feature of *N*-oxide moiety effectively avoided undesired RBC internalization. With this discovery, they successfully delivered 7-Ethyl-10-hydroxycamptothecin (SN38) to tumor tissues by hitchhiking RBCs and significantly enhanced tumor suppression in HepG2 tumor-bearing mice models 82.

#### Site-specific adsorption

The presence of abundant receptors on the cell surface provides excellent opportunities for nanomaterials to hitchhike circulatory cells through ligand-receptor interactions 52, 83, 84. The CD44, which is widely expressed on macrophages, T cells, B cells, and tumor cells, has been extensively utilized in facilitating the attachment of nanomaterials to various cell types 85-87. The interaction between hyaluronic acid (HA) and CD44 has been particularly effective in this regard, as demonstrated by Rubner *et al.*, who successfully anchored HA-decorated patches to T cells and B cells through the HA-CD44 interaction 88. Similarly, glycophorin A (GPA), a sialo-glycoprotein found on the surface of red blood cells (RBCs), has been targeted for hitchhiking purposes. For example, Brenner *et al.* achieved efficient hitchhiking of RBCs by utilizing anti-GPA antibodies and the GPA receptor for specific binding 89. In addition to ligand-receptor interactions, a positive targeting strategy can also be employed to hitchhike circulatory cells. This approach initially anchors affinity ligands such as biotin, aptamers, antibodies, or peptides onto the cell membranes 90. Subsequently, nanomaterials can be site-specifically adsorbed onto the cells by interacting with these ligands and their corresponding receptors 91. This strategy allows for calculating the extent of conjugation based on the surface densities of membrane proteins, ensuring precise control over the hitchhiking process while minimizing the risk of compromising the surface properties of the hitchhiking cells.

The adsorption of nanoparticles to cells holds great promise for targeted drug delivery and imaging in nanomedicine applications. However, to ensure the safe and effective use of this approach, it is crucial to thoroughly investigate its impact on cellular functions, particularly cell migration 22, 92. Several concerns regarding cellular hitchhiking need to be addressed: (1) Cellular functionality: the attachment of materials or nanoparticles may affect the functionality of surface proteins on the cell membrane, potentially altering the cell's ability to interact with its environment and respond to external stimuli. This could have unintended consequences on cell behavior, potentially compromising the intended applications of hitchhiked cells; (2) Immune response: modified cells may provoke an immune response from the body, leading to potential rejection or inflammatory reactions. Even if the attached materials are designed to be biocompatible, the immune system might still recognize the hitchhiked cells as foreign entities; (3) Long-term effects: the long-term consequences of cellular hitchhiking are not yet fully understood. It is vital to investigate whether the attached materials degrade over time or accumulate on the cell surface, which could potentially lead to adverse effects on cell health. To make cellular hitchhiking a viable and safe approach, researchers must carefully design and characterize the materials used for hitchhiking. Thorough biocompatibility testing, *in vitro* and *in vivo* studies, and comprehensive analysis of cellular responses are essential steps to understand the impacts on cell function. Biodegradable and non-toxic materials are preferred to ensure that they do not accumulate in the body and cause harm over time. In this regard, nanoparticles with surface coatings that enhance biocompatibility and reduce immune responses are being explored to address these issues. Moreover, researchers must establish clear guidelines and safety standards for the clinical use of hitchhiked cells. Thorough preclinical studies and rigorous risk evaluation are essential prerequisites before translating cellular hitchhiking techniques into human therapies. In conclusion, cellular hitchhiking offers exciting possibilities in biomedical applications, but the biocompatibility of attached materials and nanoparticles must be carefully evaluated. Understanding the tradeoffs and potential risks associated with cellular hitchhiking is essential to harness its potential benefits while ensuring the safety and effectiveness of this innovative approach.

## Cell/protein choices for *in vivo* hitchhiking

The circulatory cells/proteins exhibit several unique properties, including unrivaled systemic circulation capabilities, inherent stealth properties, natural delivery mechanisms, and the ability to navigate and traverse biologically impermeable barriers, which make circulatory cells/proteins ideal vehicles for targeted drug delivery. These features arise from the mechanical properties, distinctive structure, and surface ligands characteristic of each specific cell type. Therefore, a comprehensive understanding of these cellular functions is a prerequisite for the development of successful cell hitchhiker formulations. In this section, we will briefly describe the structure and functions of three major cells/proteins for *in vivo* hitchhiking, including neutrophiles, RBCs, and serum albumin.

### Hitchhiking on neutrophils

Neutrophils, also referred to as polymorphonuclear leukocytes, heterophils, or neutrocytes, represent the predominant class of granulocytes, comprising approximately 40-70 % of all WBCs in humans 93, 94. The neutrophils play a crucial role as the frontline defense in human immunity, rapidly activating in response to inflammatory stimuli 95. This activation triggers an up-regulation of surface receptors, such as CD11b, CD14, and TLR 4, augmenting their phagocytic capability 96-98. Additionally, neutrophils demonstrate an impressive tropism for inflamed regions, enabling them to traverse various biological barriers, including blood vessels and the blood-brain barrier (BBB), through deformation and other mechanisms 99, 100. Upon reaching the inflammation site, neutrophils release neutrophil extracellular traps (NETs), facilitating the traceless release of hitchhiking nanomaterials 101. Based on these unique characteristics, several neutrophile hitchhiking nanomaterials have been developed 57, 63, 102-105. For example, Wang *et al.* presented a piceatannol (spleen tyrosine kinase inhibitor)-loaded denatured BSA nanoparticles that can efficiently disrupt β2 integrin signaling in leukocytes by hitchhiking neutrophils and mitigate vascular inflammation 56. Employing a similar approach, they successfully achieved selective elimination of inflammatory neutrophils and alleviated neurological damage in stroke through systemic administration of doxorubicin (DOX)-loaded BSA nanoparticles 103. Another notable contribution by Wang *et al.* involved the presentation of a specific lipid, DOCP, which rapidly forms a protein corona containing complement fragment iC3b 57, 106, 107. This corona facilitated neutrophil hitchhiking through C3 receptor-mediated phagocytosis. Subsequently, the hitchhiked neutrophils were able to carry liposomes to migrate across the alveolar-capillary barrier into inflamed tissues and release ampicillin-loaded liposomes via the formation of NETs, resulting in a significant alleviation in acute lung injury (ALI). Similarly, Liu *et al.* presented that another lipid, DOPG, exhibited a similar behavior to DOCP that can selectively adsorb complement fragment iC3b to facilitate neutrophil hitchhike 105. By systemically administering a DOPG-modified NIR-IIb emissive immunotracer, they successfully achieved dynamic monitoring of neutrophils in inflammation-related diseases. Although promising, neutrophil hitchhiking may also cause some problems related to toxicity and bioaccumulation. Toxicity concerns: Neutrophils are immune cells that release ROS and cytotoxic enzymes to destroy pathogens. The hitchhiking process or the cargo itself might interfere with the normal function of neutrophils, affecting their ability to combat infections and potentially leading to immune-related toxicity. Bioaccumulation: After internalization, the premature release of loaded drugs in neutrophils may impair their migration, homing, and other functions, leading to unpredictable therapeutic efficacy. Therefore, precise control of the drug release profile and avoiding undesired interference with neutrophils are crucial factors in successfully hitchhiking on neutrophils.

### Hitchhiking on RBCs

Red blood cells (RBCs), or erythrocytes, have been widely recognized and utilized as highly established and popular natural carriers for drug delivery since the 1970s. This is primarily due to their remarkable and diverse advantages 108. For example, RBCs have an extremely long circulating half-life of approximately 120 days in humans, exhibit excellent biocompatibility and accessibility, and can be utterly degraded without producing toxic metabolites 109. Moreover, RBCs possess a distinct biconcave shape with great natural markers on their surface, including glycan, sialic acid, protein, and CD47, offering numerous opportunities for efficient drug loading and *in vivo* hitchhiking 110, 111. Based on these unique characteristics, several RBCs hitchhiking nanomaterials have been developed. For example, Brenner *et al.* presented a dual affinity to RBCs and target cells (DART) strategy for highly efficient organ and cell-type targeting 89. DART strategy was achieved by conjugating two types of antibodies that selective binding to RBCs (anti-GPA) and endothelial epitopes (anti-PECAM) onto liposomes. After intravenous injection, the DART nanocarriers efficiently hitchhiked RBCs through GPA/anti-GPA interaction and selectively bound to the pulmonary vasculature under the guidance of RBCs. Similarly, Ranjan *et al.* proposed a Ter119 antibody-modified polymer nanoparticle and efficiently hitchhiked erythrocytes by targeting GPA on erythrocytes, thereby significantly prolonging the circulation time of the nanoparticles without causing any histological, hematological, and morphological complications 112. In addition to antigen-antibody interactions, amphiphilic molecules are reported to exhibit binding affinities toward RBCs. For example, Brooks *et al.* demonstrated that amphiphilic polyethylene glycol (PEG)-hyperbranched polyglycerol (HPG)-polymers incorporating stearoyl chains (HPG-C18-PEG) were capable of effective adsorption onto RBCs 113. Similarly, Shen *et al.* presented that zwitterionic liposomes containing tertiary amine oxide (TAO) achieved efficient hitchhiking on RBCs 82. Furthermore, a host-guest strategy has also been employed for RBCs hitchhiking. For example, Wang *et al.* reported a host-guest strategy that exploits the interaction between β-cyclodextrin (β-CD) and ferrocene (Fc) to facilitate the binding of liposomes to RBCs 114. The researchers initially modified RBCs with DSPE-PEG-CD and integrated Fc groups into liposomes, forming RBCs-CD and Lipo-Fc, respectively. Through the host-guest interactions between β-CD and Fc, Lipo-Fc effectively hitchhiked onto RBCs-CD, significantly improving circulation stability. This RBCs-hitchhiking strategy offers significant advantages regarding prolonged circulation and controlled release of therapeutic enzymes, potentially benefiting scenarios like alcohol poisoning. Moreover, it holds promise for delivering nanoparticles to body regions with high cardiac output, allowing targeted delivery of therapeutic nanoparticles to adjacent pathologies.

Despite the significant successes achieved with RBCs-hitchhiking, some limitations should be considered. Toxicity concerns: RBCs are generally considered inert and typically do not induce significant immune responses or toxicity in the body. However, safety concerns may arise, particularly hemolysis, which could occur during the hitchhiking process or following delivery to the target site. The rupture of RBCs may lead to the release of hemoglobin and other cellular contents, potentially causing adverse reactions. Bioaccumulation: RBCs have a relatively short lifespan of approximately 120 days in circulation and are eventually cleared from the body through the spleen and liver. Nonetheless, in cases where the nanoparticles remain within hitchhiked RBCs for an extended period, there exists a potential risk of bioaccumulation. This could result in the gradual build-up of cargo over time, leading to unintended consequences. Therefore, careful consideration and comprehensive evaluation of such approaches are necessary to ensure their safety and effectiveness in biomedical applications.

### Hitchhiking on serum albumin

Serum albumin (SA), with a molecular weight of 66.5 kDa, is the most abundant protein found in the bloodstream 115. It possesses a remarkable systemic circulation time of approximately 19 days 116. In addition, SA exhibits a distinctive pocket structure that enables the transportation of hydrophobic molecules, including metal ions, drugs, long-chain fatty acids, and hormones, throughout the bloodstream 117. Furthermore, SA also exhibits remarkable capabilities in diverse physiological processes, such as evasion of renal clearance, transcytosis across endothelial cells, and efficient accumulation in rapidly growing nutrient-poor tissues 118. These unique features make SA ideal candidate for *in vivo* hitchhiking, offering significant potential for optimizing therapeutic outcomes. A general strategy for *in situ* SA hitchhiking involves the use of maleimides for covalent attachment to thiol groups on SA cysteines. Notably, this thiol group is the most abundant free thiol in blood, while other thiol functional groups usually exist in the form of non-reactive disulfide bonds, effectively avoiding undesired off-target reactions 119. For example, Liu *et al.* conjugated maleimide to paclitaxel (PTX) and effectively promoted the accumulation of the PTX prodrugs in tumor tissues through albumin receptor-mediated active targeting 120. Similarly, Unger *et al.* presented a maleimide-conjugated doxorubicin for SA hitchhiking, demonstrating significantly enhanced tumor suppressions in breast carcinoma MDA-MB 435 and breast carcinoma MCF-7-bearing mice models 119. In addition to the maleimide group, the meso-chloro is another chemical moiety capable of reacting with the thiol group of albumins. For example, Wang *et al.* reported a meso-chloro-integrated near-infrared window II (NIR-II) fluorescence probe, IR1080, which demonstrated precise detection of micro-metastases and facilitated fluorescence image-guided surgery through SA hitchhiking 121.

In addition to the covalent-based strategy for SA hitchhiking, the unique pocket structure also allows nanomaterials to hitchhike onto SA through hydrophobic or other interactions. For example, Irvine *et al.* discovered that lipo-PEG amphiphiles with two hydrophobic tails could efficiently insert into the hydrophobic core of albumin through hydrophobic interactions 122. They further investigated the impact of hydrophobic tail length on the efficiency of albumin insertion and observed that longer diacyl tails (≥ 16 carbons) exhibited a higher affinity for albumin. With this finding, they successfully utilized SA hitchhiking to deliver lipo-CpG to lymph nodes, resulting in significantly enhanced T cell priming and cancer immunotherapy. Similarly, Tan *et al.* employed this strategy to achieve tumor-targeted delivery of a floxuridine homomeric oligonucleotide (LFU20) for enhanced cancer chemotherapy 123. Another frequently used functional group for SA hitchhiking is sulfonated azophenyl, which exhibit strong binding ability towards SA. For example, Evans Blue (EB), which contains two sulfonated azophenyl groups, is a commonly used SA-specific azo dye 124, 125. After systemic administration, EB selectively binds to circulating SA and assesses BBB permeability. Furthermore, several EB derivatives have been developed for long-acting therapeutics and diagnostic imaging. For example, diethylenetriaminepentaacetic acid-conjugated truncated EB (EB-DTPA) for gadolinium (III) complexation and magnetic resonance imaging (MRI) 126; 1,4,7-triazacyclononane-*N,N',N''*-triacetic acid (NOTA) conjugated truncated EB (NEB) for isotopes labeling (^68^Ga, ^64^Cu, and Al^18^F) and positron emission tomography (PET) imaging 127; EB-maleimide conjugated exendin-4 for significantly enhanced circulation stability and type 2 diabetes/obesity treatment 128. Moreover, EB analogs have also been introduced to macrocyclic hosts. For example, Guo *et al.* presented a sulfonated azocalix[5]arene (SAC5A) for tumor-targeted delivery of paclitaxel (PTX) 9. SAC5A efficiently loads PTX *in vitro* through host-guest interaction to form a binary supramolecular prodrug (PTX@SAC5A). Following intravenous injection, PTX@SAC5A rapidly hitchhikes with circulating SA to form a ternary formulation, PTX@SAC5A/albumin, which significantly prolongs the circulating half-life of PTX and dramatically improves its antitumor efficacy.

Serum albumin is a protein present in the bloodstream and is widely considered safe for therapeutic applications due to its natural occurrence in the body. Nevertheless, there are potential concerns regarding toxicity if the hitchhiked cargo interacts with albumin in a manner that disrupts its normal physiological function or results in unintended effects. Moreover, serum albumin exhibits a relatively extended half-life (approximately 20 days), making it an appealing carrier for sustained delivery of therapeutic payloads. However, this prolonged circulation time also raises the possibility of bioaccumulation if the hitchhiked cargo is not efficiently released from the albumin molecule. To address these challenges and optimize the therapeutic benefits, it is crucial to meticulously design and optimize the hitchhiking process and release mechanisms.

### Hitchhiking on platelet

Platelets, also known as thrombocytes, are small, disc-shaped blood cells present in mammals' bloodstream, including humans. Like neutrophils, platelet can efficiently accumulate in injured or inflamed tissues and activated in response to inflammatory stimuli 129. This activation triggers an up-regulation of surface receptors, such as P-selectin, GPIb-V-IX, and integrin aIIbb3 (GPIIb/IIIa) 130. These receptors allow the effective binding of activated platelets on circulating tumor cells (CTCs) or injured tissues, enabling them to carry out essential functions like tumor metastasis, tissue repair, or hemostasis. Given the specific binding capabilities to these receptors, nanoparticles can be designed to adsorb on platelets and hitchhike along with them, facilitating the delivery of anticancer or antithrombosis drugs. For example, He *et al.* introduced a fucoidan-decorated DOX-loaded polymeric micelle for the treatment of metastatic cancer 131. Within the bloodstream, the fucoidan groups of the micelle demonstrated exceptional adhesion to activated platelets on circulating tumor cells (CTCs) through fucoidan/P-selectin interaction 132, 133, enabling effective hitchhiking of platelets and their accumulation in tumor tissues. Consequently, the DOX efficiently induced immunogenic cell death (ICD), showcasing highly effective tumor suppression in a 4T1 tumor mice model. This study highlights the potential of platelet-mediated hitchhiking as a promising strategy for targeted drug delivery and improved cancer therapy outcomes.

## Biomedical applications of hitchhiking nanomaterials

By hitchhiking on circulatory cells or proteins, these rationally designed nanomaterials exhibit multiple advantages over conventional carriers, including significantly enhanced circulation stability and half-life, active targeting, and minimized off-target effect. Benefiting from these unique properties, hitchhiking nanomaterials are increasingly used in biomedical applications. This section provides a comprehensive overview of recent advancements in hitchhiking nanomaterials for ischemic stroke, acute lung injury, cancer treatment, gene therapy, and diagnostic imaging.

### Neutrophils-hitchhiking nanomaterials for ischemic stroke treatment

**Figure 2:**
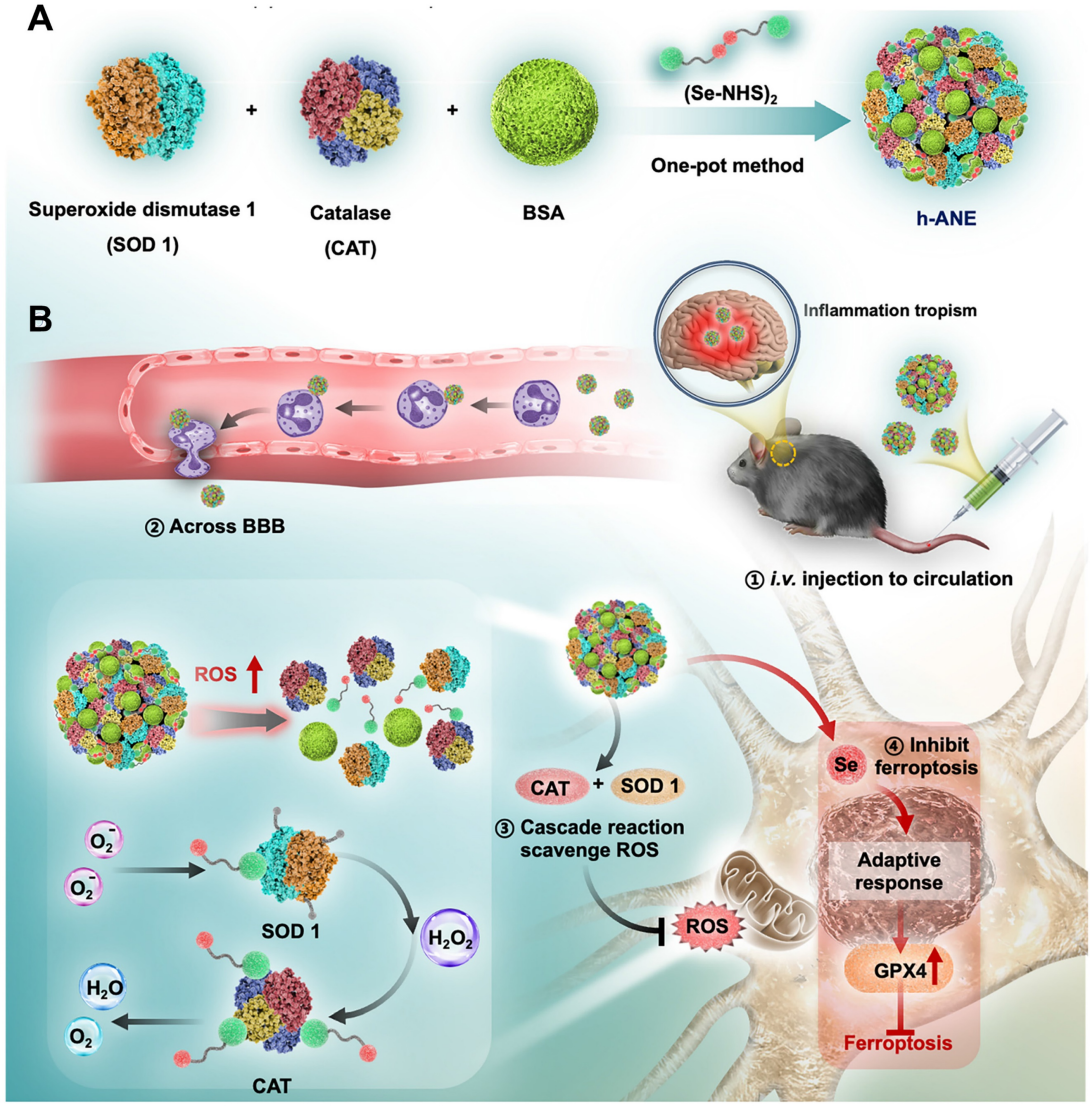
Schematic illustrating the “neutrophil piggybacking” h-ANEs for antioxidant and anti-ferroptosis therapy of cerebral I/R injury. **A**, Schematic illustration of the preparation of h-ANE. **B**, The biological process of h-ANEs for antioxidant and anti-ferroptosis therapy of cerebral I/R injury. Adapted with permission from 135, copyright 2022 Wiley-VCH.

Ischemic stroke is a fatal disease caused by insufficient blood supply to the brain, often accompanied by an accumulation of toxic reactive oxygen species (ROS) in the brain. Effective treatment of ischemic stroke necessitates addressing two key aspects: (1) crossing the BBB and (2) eliminating ROS. The inflammatory tropism of neutrophils makes hitchhiking neutrophils an ideal strategy to cross the BBB and treat ischemic stroke 100, 134. For example, Liu *et al.* presented nano-integrated cascade enzymes (h-ANEs) composed of superoxide dismutase (SOD), peroxidase (CAT), and BSA (**Figure 2**) 135. In the bloodstream, the integrated albumin exhibits excellent adhesion to activated neutrophils, enabling effective hitchhiking of neutrophils to cross the BBB and accumulate in cerebral infarction areas. Subsequently, the SOD and CAT of h-ANEs efficiently eliminate toxic ROS, demonstrating highly efficient neuroprotection in an ischemia-reperfusion injury mouse model. Tang *et al.* reported a PLGA-PEG-based polymeric nanoparticle (T-TMP) to deliver ligustrazine to treat cerebral ischemia-reperfusion injury 52. The surface of T-TMP is integrated with a neutrophils-targeting peptide (CFLFLF), which can effectively direct T-TMP to neutrophils and thus hitchhike neutrophils after intravenous injection. Upon reaching lesion tissues, T-TMP rapidly responds to high levels of ROS and release ligustrazine, resulting in significantly enhanced neuroprotection. Similarly, Zhou *et al.* leveraged the bacteria-scavenging capabilities of neutrophils and developed a bacteria-derived outer-membrane vesicle (OMV) for targeted delivery of pioglitazone (PGZ) to the brain 84. In addition to eliminating ROS, inhibiting the infiltration of inflammatory cells in the brain is also an effective way to relieve ischemic stroke. For example, Wang *et al.* presented a DOX-conjugated BSA nanoparticle capable of selectively inducing neutrophil apoptosis, effectively preventing brain injury in cerebral ischemia/reperfusion mice model 103. Overall, these innovative strategies that leverage neutrophils' inflammatory tropism and scavenging abilities hold great promise for developing targeted and effective therapies for ischemic stroke. By addressing both ROS accumulation and inflammatory cell infiltration, these approaches open up new possibilities for enhancing neuroprotection and improving outcomes for stroke patients. Further research and clinical studies will be essential to validate and optimize these techniques for eventual translation into clinical practice.

### Neutrophils-hitchhiking nanomaterials for acute lung injury (ALI) treatment

ALI is a diffuse heterogeneous lung injury characterized by widespread capillary leakage, low lung compliance, non-cardiogenic pulmonary edema, and hypoxemia 136, 137. In ALI, the lungs experience a significant influx of inflammatory cells, predominantly neutrophils, into the affected areas 138. This influx of inflammatory cells plays a critical role in exacerbating lung injury and contributing to the overall pathology of ALI. The underlying pathology of ALI is largely attributed to the cytokine storm triggered by the NF-*κ*B pathway. The NF-*κ*B pathway is a central regulator of inflammation and immune responses in the body 139. Its dysregulation leads to the overproduction of pro-inflammatory cytokines and chemokines, causing an excessive lung inflammatory response. This, in turn, damages the lung capillaries, leading to the observed capillary leakage and pulmonary edema in ALI. Hypoxemia, a condition characterized by low levels of oxygen in the blood, is a prominent consequence of ALI and contributes to the severity of the disease. Due to the critical role of the NF-*κ*B pathway in driving the cytokine storm and subsequent lung inflammation, researchers have identified it as a promising therapeutic target for ALI treatment. For example, Wang *et al.* developed a novel approach using denatured BSA nanoparticles (BSA-NPs) as a drug delivery system to specifically target the lungs and deliver an NF-*κ*B inhibitor, TPCA-1 102. In their study, these BSA-NPs were designed to take advantage of the innate ability of neutrophils to home in on inflamed tissues. When administered intravenously, the BSA-NPs are selectively internalized by neutrophils and subsequently accumulate in the injured lung tissues. By delivering the NF-*κ*B inhibitor directly to the site of inflammation, this targeted approach effectively reduces the activation of the NF-*κ*B pathway. As a result, there is a significant decrease in lung inflammation, leading to alleviating ALI symptoms and improved lung function. This innovative approach of using denatured BSA nanoparticles for targeted drug delivery not only enhances the therapeutic efficacy of the NF-κB inhibitor but also minimizes off-target effects on other organs, making it a safer and more efficient strategy for ALI treatment.

**Figure 3:**
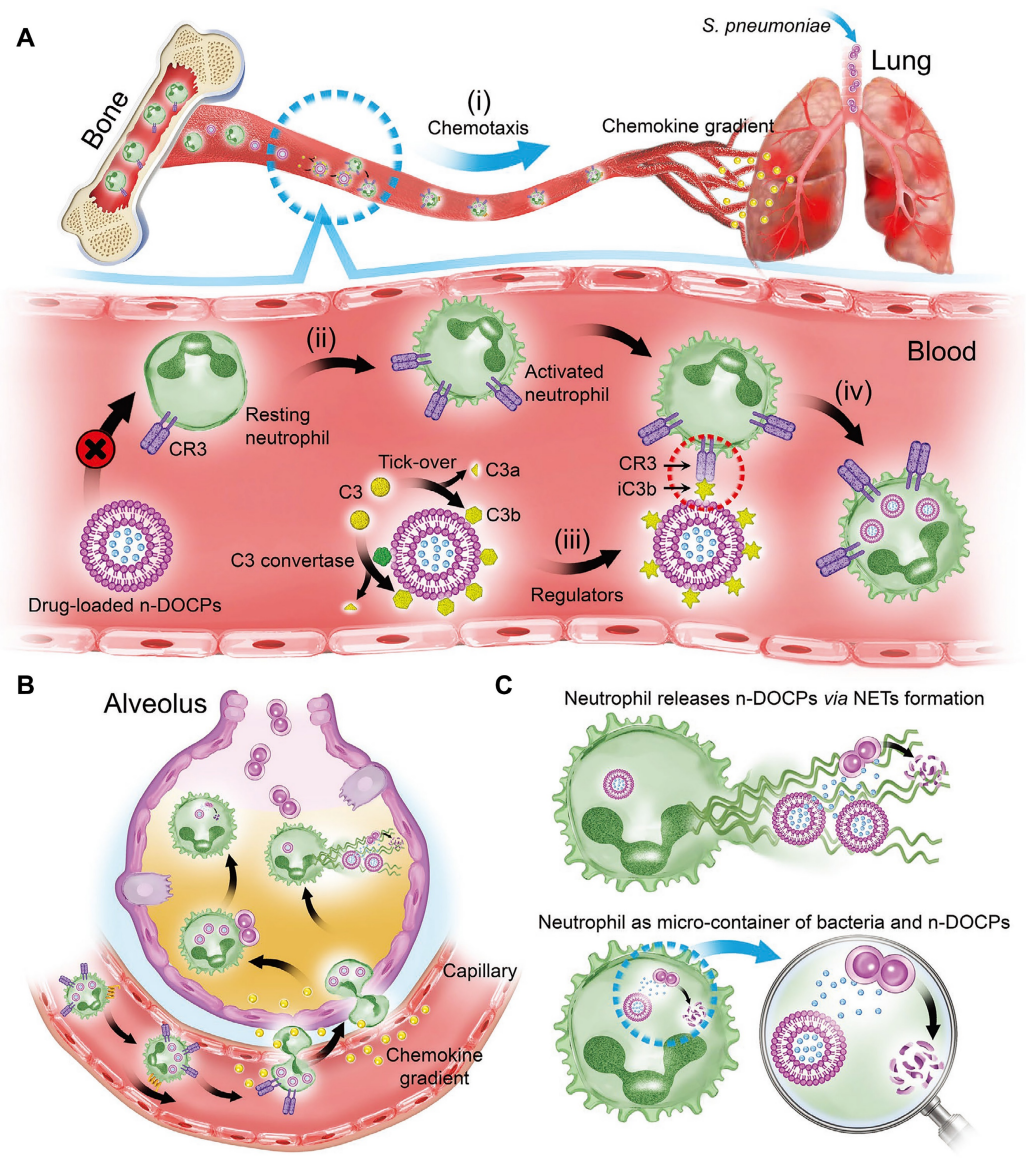
Schematic illustration of complement fragments tagging on the nanoparticles mediated rapid neutrophilic entry for inflammation targeting. **A**, *In vivo* hitchhiking process of n-DOCPs on activated neutrophils. **B,** The hitchhiked neutrophils move to inflammation area under the motivation of chemokine gradient. **C,** Neutrophils release n-DOCPs for enhanced anti-inflammation treatment. Adapted with permission from 57, copyright 2021 Wiley-VCH.

Besides the NF-*κ*B pathway, several clinical studies also suggest that endoplasmic reticulum stress (ER stress)-induced macrophage polarization contributed to the development of ALI. ER stress is a condition that occurs when the endoplasmic reticulum, a cellular organelle involved in protein synthesis and folding, becomes overwhelmed or damaged, leading to the accumulation of misfolded proteins. This stress triggers a response known as the unfolded protein response (UPR), which aims to restore ER homeostasis. However, if the stress persists or is too severe, it can lead to macrophage polarization, which contributes to the pathogenesis of ALI. Relieving ER stress to restore macrophage homeostasis represents a potential approach for ALI prevention. To this end, You *et al.* presented HA-decorated multistage targeted nano-micelles for delivering ER stress inhibitor (KIRA6) and anti-inflammatory drugs dexamethasone (Dex) to infected lungs 140. In the bloodstream, the nano-micelles effectively hitchhike on myeloid macrophages and neutrophils through CD44/HA interaction, thus effectively accumulating in infected lungs. Subsequently, the drug-loaded nanoparticle quickly responds to high-level ROS to facilitate the release of KIRA6 and Dex, thereby effectively relieving ER stress, balancing macrophage homeostasis, and finally resolving ALI. Additionally, delivering anti-inflammatory drugs or antibiotics to the lung is feasible for ALI treatment. For example, Wang *et al.* found that liposomes comprising inverted phosphocholine lipids rapidly enriched iC3b through voluntary opsonization, facilitating neutrophil hitchhiking through C3 receptor-mediated phagocytosis (**Figure 3**) 57. With this strategy, they effectively delivered Dex and antibiotics ampicillin (Amp) to the lung, leading to a significant attenuation of lung inflammation. These strategies underscore the importance of exploring innovative approaches for ALI prevention and treatment, especially those that leverage the innate properties of immune cells for targeted drug delivery. These studies offer great potential for more effective and tailored therapies that may improve patient outcomes and reduce the burden of this severe and life-threatening condition.

### Hitchhiking nanomaterials for cancer treatment

**Figure 4:**
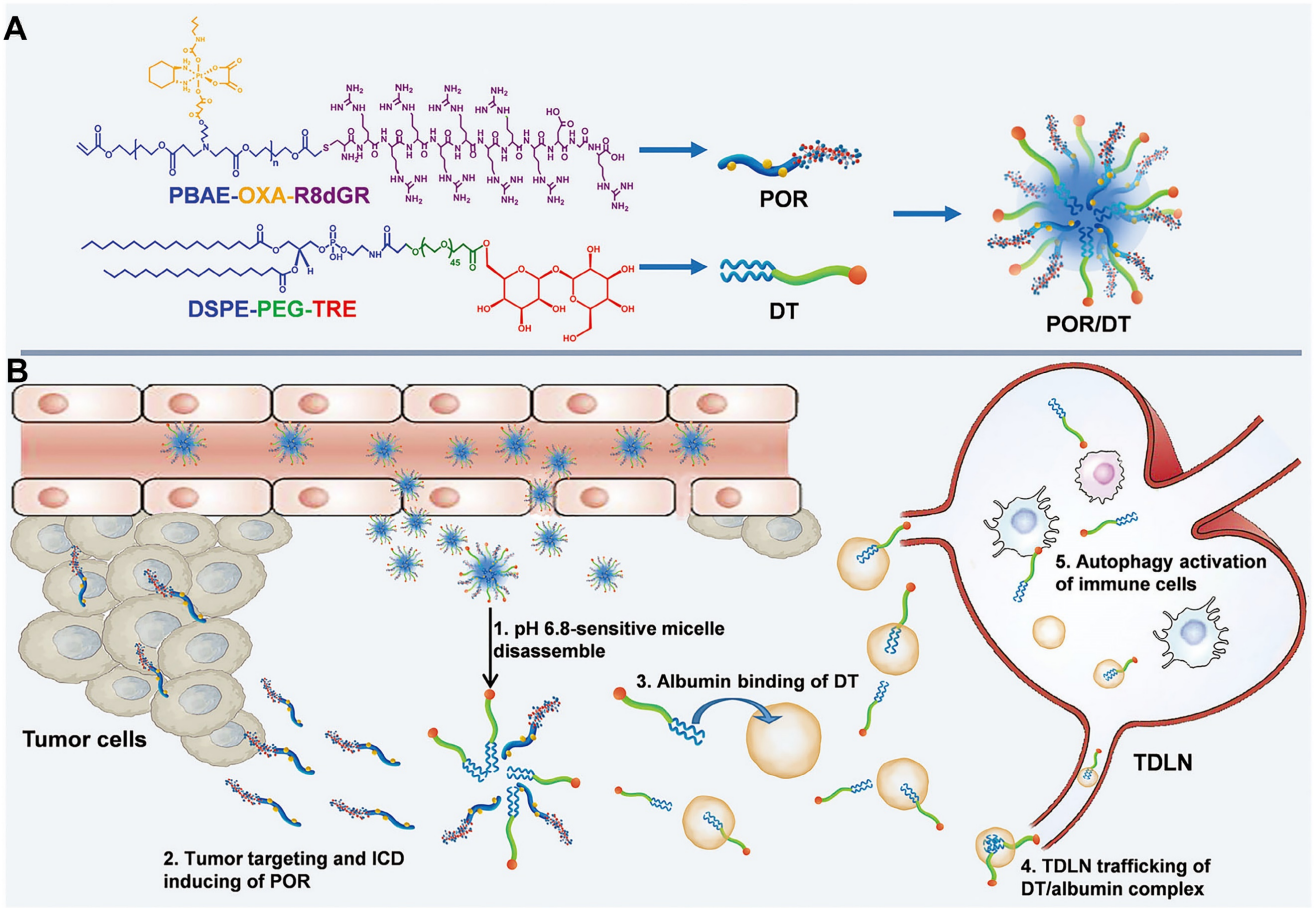
Schematic illustration of the albumin hitchhiking nanomedicine for tumor immunotherapy by autophagy activation and ICD induction. **A,** The preparation of POR/DT. **B,** The biological process of POR/DT for enhanced cancer immunotherapy. Adapted with permission from 144, copyright 2021 Wiley-VCH.

Cancer is a leading cause of death worldwide, characterized by rapid proliferation and high invasiveness 141. Multiple genetic mutations and alterations lead to a unique tumor microenvironment (TME) and the upregulation of tumor-specific biomarkers 142. For example, GP60 and secreted protein acidic and rich in cysteine (SPARC) are albumin-binding proteins highly expressed in various tumors 143. Exploiting the hitchhiking strategy on serum albumin offers a promising approach for effective cancer treatment. First, albumin is abundant in the bloodstream and is known to accumulate in the tumor due to the EPR effect. This passive targeting of the tumor by albumin carriers increases the drug's concentration at the tumor site while reducing systemic exposure and potential side effects. Additionally, the hitchhiking approach takes advantage of tumor-specific biomarkers like GP60, ensuring preferential uptake and internalization of the drug-loaded complex by cancer cells, further enhancing treatment efficacy 143. Tan *et al.* presented a lipid-conjugated floxuridine oligonucleotide (LFU20) that hitchhikes SA to enhance cancer chemotherapy 123. In blood circulation, LFU20 readily interacts with SA through hydrophobic interaction to form an LFU20/albumin complex, which then accumulates in the tumor and internalizes by cancer cells through BSA/GP60 interaction, resulting in significantly enhanced antitumor efficacy. Moreover, the small size of SA enables SA-hitchhiking materials to accumulate in lymph nodes through interstitial flow. For example, He *et al.* presented hitchhiking hybrid micelles (POR/DT) for the sequential delivery of oxaliplatin (OXA) and trehalose (TRE) to tumors and tumor-draining lymph nodes (TDLNs) (**Figure 4**) 144. POR/DT was prepared by co-assembling poly (β-amino esters)-based pH responsive polymer (POR) and DSPE-PEG-TRE (DT). Upon reaching tumor tissue, POR/DT quickly responds to acidic TME and facilitates the disintegration of POR/DT into POR and DT. Subsequently, the POR prodrug effectively targeted tumor tissues via integrin receptor recognition, while DT hitchhiked on SA and accumulated in TDLNs through interstitial flow. Finally, these two drugs synergistically activated immunogenic cell death (ICD)-associated antitumor immunity and significantly prolonged the survival rate of CT26-bearing mice. These albumin-hitchhiking approaches hold great promise for personalized and effective cancer therapies, providing a more tailored treatment approach based on the unique characteristics of each patient's tumor microenvironment and tumor-specific biomarkers.

**Figure 5:**
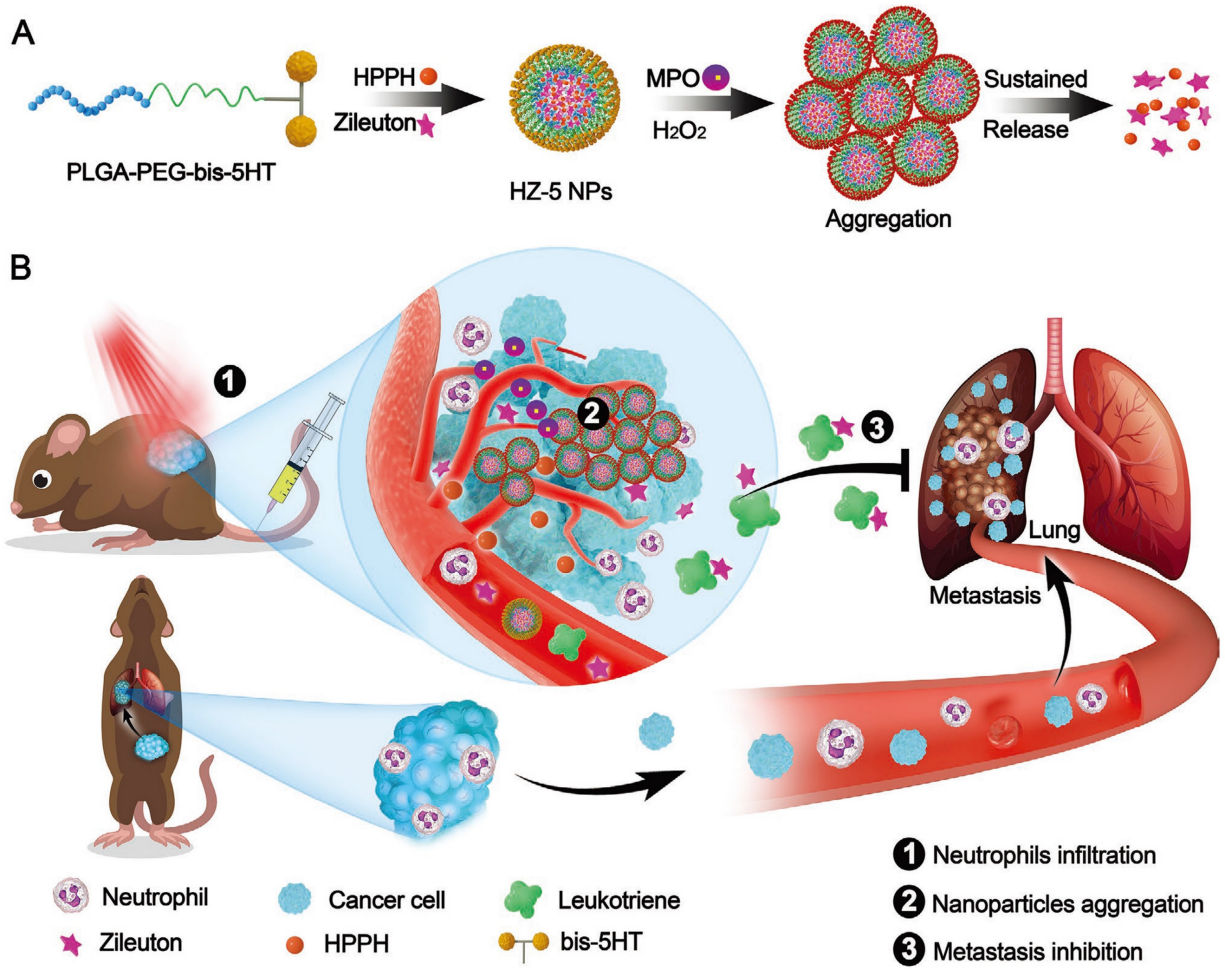
5-Hydroxytryptamine (5-HT) equipped nanoparticles (HZ-5 NPs) for enhanced cancer treatment. **A**, Design of HZ-5 NPs for MPO-catalyzed aggregation and sustained drug release. **B,** HZ-5 NPs accumulate in tumor sites and release Zileuton for enhanced cancer treatment. Adapted with permission from 145, copyright 2020 Wiely-VCH.

In addition, hitchhiking on red blood cells presents significant opportunities for enhancing the circulation half-life and stability of therapeutic agents. For example, Shen *et al.* presented a poly(2-(*N*-oxide-*N*,*N*-diethylamino)ethyl methacrylate) (OPDEA)-based SN38 conjugate for effective tumor extravasation and penetration 81. During blood circulations, the *N*-oxide moiety of OPDEA binds reversibly to the hydrophilic head of phosphatidylethanolamine and phosphatidylcholine on the RBCs membrane. Upon reaching the tumor tissues, OPDEA disassociated from RBCs, efficiently binds to tumor capillary endothelial cells (tECs) and facilitates adsorption-mediated transcytosis. This well-designed hitchhiking strategy enables the successful delivery of SN38 to deeper tumor tissues, resulting in significantly enhanced antitumor efficacy of SN38 in HepG2 tumor-bearing mice. Similarly, they also developed a series of tertiary amine oxide (TAO)-integrated liposomes and effectively delivered the SN38/CPT-11 to deeper tumors through hitchhiking on RBCs and adsorption-mediated transcytosis 82. Moreover, the inflammatory TME enables nanomaterials to hitchhike on neutrophils and macrophages for tumor-targeted drug delivery. For example, Chen *et al.* presented 5-hydroxytryptamine (5-HT) equipped nanoparticles (HZ-5 NPs) for the tumor-targeted delivery of photosensitizers (HPPH) and leukotriene inhibitor (Zileuton) to suppress tumor growth and inhibit lung metastasis (**Figure 5**) 145. In the bloodstream, the 5-HT of HZ-5 NPs efficiently targets neutrophil myeloperoxidase (MPO) and accumulates in tumor tissues through neutrophil hitchhiking. Consequently, Zileuton is released from HZ-5 NPs to inhibit leukotriene release, effectively preventing the recruitment of neutrophils into the lungs and finally inhibiting lung metastasis. Similarly, Lv *et al.* presented an anti-CD11b conjugated BSA nanoparticle (ANP) to hitchhike activated neutrophils through CD11b/anti-CD11b interaction 146. After hitchhiking, this rationally designed ANP effectively delivered decitabine (DAC) and IR820 to tumor tissues, thereby effective activating pyroptosis and significantly enhancing cancer immunotherapy. These studies represent a paradigm shift in drug delivery approaches, providing personalized and efficient cancer therapies based on the unique characteristics of each patient's tumor microenvironment and immune response.

Moreover, leveraging hitchhiking to engineer immune cells holds great promise for significantly enhancing cancer immunotherapy. For example, T cells are at the forefront of this approach, which is important in recognizing and eliminating various “enemies” such as infections, tumors, and foreign bodies 147. Nevertheless, the reduced expression or absence of T cell-targeted ligands on the tumor surface can lead to immune escape and impair antitumor efficacy. To address these issues, a promising strategy involves genetically engineering T cells with cancer-specific chimeric antigen receptors (CARs). A seminal example presented by Stephan *et al.* is that they developed an anti-CD3e f(ab')2-decorated synthetic DNA nanocarrier to *in situ* programming leukemia-specific T cells 62. This nanocarrier was loaded with plasmid DNA (pDNA) encoding leukemia-specific 194-1BBz CAR. After intravenous injection, the DNA nanocarrier efficiently targets to T cells through the CD3/anti-CD3 interaction. Subsequently, the leukemia-targeting CAR antibodies were expressed and transmembrane transported to T cells' surface, resulting in the effective recognition of leukemia cells and significantly enhanced antitumor efficacy. In addition to T cells, tumor-associated macrophages (TAMs) are another essential immune cell predominantly found in tumor tissues. They significantly contribute to the formation of immunosuppressive TME and play an essential role in promoting tumor recurrence. Jiang *et al.* assume that genetically engineering luminal TAMs with tumor-specific CARs can redirect their phagocytic ability toward tumor cells 148. By acting as APCs, these modified TAMs stimulate adaptive immune responses to prevent postoperative tumor recurrence. As a proof of concept, Jiang *et al.* presented a nanoporter to engineering macrophages/microglia for postoperative glioblastoma therapy. The nanoporter has a core-shell structure in which pDNA encoding glioma stem cells (GSCs)-specific antibody (CD133) was encapsulated inside the core with a citraconic anhydride-modified dextran (CA-dextran)-based network layer. Similar to the DNA nanocarrier reported by Stephan *et al.*, this nanoporter efficiently targets TAMs through dextran/CD206 interaction in tumor tissues, leading to the generation of CD133 overexpressed CAR-M. Subsequently, the newly generated CAR-M effectively seeks and engulfs GSCs, clearing residual GSCs and significantly enhancing antitumor efficacy. Consequently, this therapeutic approach greatly inhibits postoperative glioma recurrence, showing promise for future cancer treatments.

These *in situ* engineering strategies for immune cells hold tremendous potential in significantly enhancing cancer immunotherapy while minimizing the need for extensive purification processes and reducing the risk of undesired toxic side effects. Despite the advancements, there are still two critical directions for further improving cancer immunotherapy: i) Enhance targeting specificity to immune cells: One key aspect to address is refining the targeting specificity of the engineered therapies toward immune cells. Ensuring precise and selective targeting of immune cells will maximize the therapeutic effect while minimizing potential off-target effects on healthy cells. Research efforts should be directed towards developing more sophisticated targeting mechanisms that precisely recognize and bind to the intended immune cell populations. ii) Improve transfection efficiency in immune cells: Another vital area of improvement lies in enhancing the transfection efficiency of genetic material into immune cells. Higher transfection efficiency will result in a greater number of successfully engineered immune cells, thereby increasing the overall efficacy of the treatment. Investigating and implementing novel transfection techniques and delivery systems could lead to more efficient and reliable gene modification of immune cells. By advancing research in these two directions, cancer immunotherapy can make significant strides in achieving safer, more effective, and targeted treatments for cancer patients.

### Hitchhiking nanomaterials for other applications

#### Genetic disease treatment

**Figure 6:**
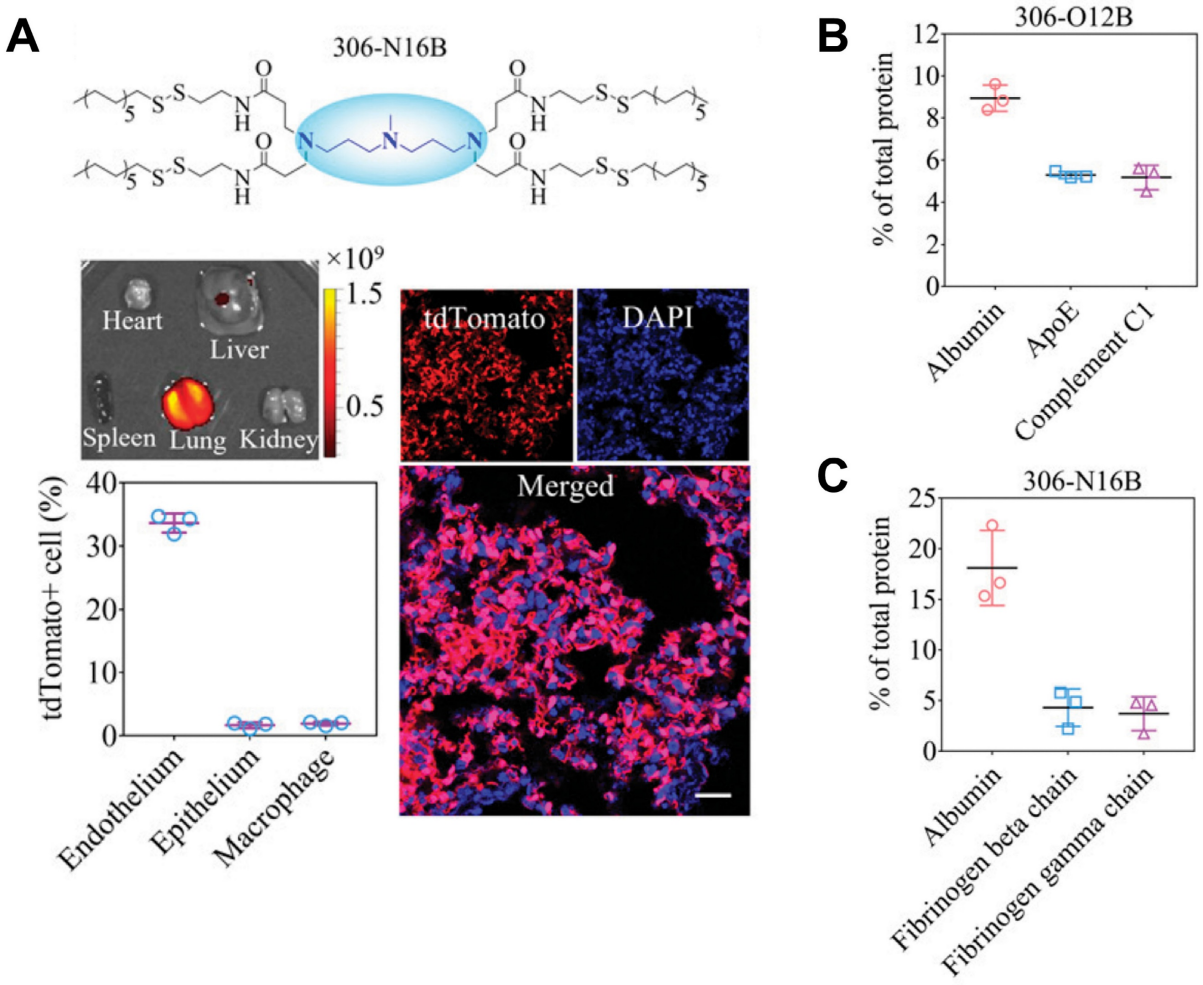
Lung-selective liposomes for pulmonary lymphangioleiomyomatosis treatment. **A**, 306-N16B specifically deliver mRNA to lung. **B**, The major component in protein corona formed by 306-O12B. **C**, The major component in protein corona formed by 306-N16B. Adapted with permission from 155, copyright 2022 National Academy of Sciences.

Gene therapy is a cutting-edge biotechnology that seeks to achieve therapeutic impacts by manipulating gene expression or altering the biology of living cells 149. Depending on the duration of action, gene therapy can be broadly categorized into two main types: permanent regulation at the genome level, or temporary regulation at the protein level. Typically, these therapeutic interventions are administrated in forms of proteins, RNA, or plasmid DNA (pDNA) and require crossing multiple physiological barriers before exerting their efficacy. To this end, multiple non-virus vectors based on cationic polymer, dendrimers, micelles, and lipid nanoparticles (LNP) are developed for gene delivery 141, 150, 151. However, these vectors are mainly distributed in the liver, and how to perform gene editing in non-liver organs remains challenging. Siegwart *et al.* presented a selective organ targeting (SORT) strategy for the selective delivery of message RNA (mRNA) to the liver, spleen, or lung 152, 153. Liver SORT, spleen SORT, and lung SORT were formulated by incorporating ionizable cationic lipid, anionic lipid, and permanently cationic lipid, into initial mRNA formulation (5A2-SC8, DOPE, cholesterol, DMG-PEG; 15/15/30/3, mol/mol), respectively. Upon intravenous injection, Liver SORT efficiently adsorbs apolipoprotein E (ApoE) and hitchhiking on ApoE to accumulated in liver through ApoE/low-density lipoprotein receptor (LDL-R) interaction. Similarly, spleen SORT and lung SORT efficiently adsorbs β2-glycoprotein I (β2-GPI) and vitronectin (Vtn) and hitchhiking on these proteins to accumulated in spleen throughβ2-GPI/phosphatidyl serine interaction or lung through Vtn/α_v_β_3_ integrin interaction, respectively. With this strategy, they effectively delivered various types of mRNA to liver, spleen, and lung, demonstrating great potential for gene editing in non-liver organs. Similarly, Xu *et al.* discovered that LNPs belonging to the O-series, which contain an ester bond in the tail, have a tendency to deliver mRNA specifically to the liver (**Figure 6**) 154, 155. Conversely, LNPs belonging to the N-series, which contain an amide bond in the tail, demonstrate the capability to selectively deliver mRNA to the lungs of mice. This organ selectivity observed with LNPs is analogous to the behavior of SORT molecules. Specifically, O-series LNPs efficiently adsorb and hitchhike apolipoprotein E (ApoE), while N-series LNPs effectively adsorb and hitchhike fibrinogen. Using this strategy, they successfully delivered mouse tuberous sclerosis complex 2 (Tsc2) mRNA to the lungs. This led to the efficient restoration of the TSC2 tumor suppressor, resulting in a remarkable therapeutic effect by reducing tumor burden. The ability to selectively deliver therapeutic agents to specific organs offers the potential to develop organ-specific therapies and personalized medicine approaches. Despite these advancements, challenges remain in optimizing gene delivery to target organs while ensuring safety and efficacy. Continued research, advancements in vector design, and further understanding of organ-specific interactions will be essential for harnessing the full potential of gene therapy and its application in clinical settings. The development of targeted gene editing approaches for diverse organs holds the promise of transforming the landscape of medicine, paving the way for more effective and personalized treatments for a wide range of diseases.

#### Alzheimer's disease treatment

Alzheimer's disease (AD) is a chronic and irreversible neurodegenerative condition with prodromal phases, a prolonged preclinical period, and an average clinical duration of 8 to 10 years 156, 157. AD pathology is marked by a progressive decline in mental, functional, and behavioral abilities, along with a loss of learning capacity. One significant hypothesis regarding AD revolves around the accumulation of cortical and cerebrovascular deposits, specifically amyloid-β (Aβ) peptide aggregates. In the pursuit of treating AD, an important strategy involves the use of nanomedicines to prevent the formation of Aβ aggregates and facilitate the removal of existing ones. In the process of AD, the circulatory serum Aβ will automatically accumulate in the brain. Hitchhiking on circulatory serum Aβ should be effective in crossing BBB and alleviating AD symptoms. For example, Gao *et al.* presented a KLVFF-decorated polydopamine nanoparticles (PDA@K) for AD treatment 158. After intravenously injection, the KLVFF of PDA@K efficiently binding to serum Aβ through hydrophobic interaction, enabling it to cross the BBB via Aβ hitchhiking and accumulate in the lesion site. Subsequently, the PDA@K effectively chelated Cu^2+^ and Zn^2+^, leading to a significant suppression of aggregate formation and the restoration of behavioral and cognitive abilities. This study provides compelling evidence to consider metal dyshomeostasis and the inflammatory microenvironment as potential therapeutic targets for AD. Such a multi-pronged strategy could offer novel approaches to explore and develop treatments for this challenging neurodegenerative disease.

#### Non-invasive diagnostic imaging

Non-invasive diagnostic imaging holds tremendous promise in facilitating early disease detection and treatment. The commonly employed techniques for *in vivo* diagnostic imaging include positron emission tomography (PET), magnetic resonance imaging (MRI), computed tomography (CT), and fluorescence imaging. These techniques typically rely on the utilization of imaging probes, such as radionuclides, metal ions, and photosensitizers, to help visualize targeted structures or pathological changes. In recent years, several innovative probes capable of hitchhiking on circulatory cells or proteins have been developed and extensively utilized. This hitchhiking strategy confers two significant advantages to these imaging probes: (1) an extended half-life and (2) significantly enhanced targeting ability to lesion tissues. For example, Liu *et al.* presented a phospholipids DOPG-incorporated lanthanide-doped nanoparticle (LnNPs@PG) that enables dynamic monitoring of neutrophils in inflammation-associated disease 105. In bloodstream, the hydroxyl groups of LnNPs@PG efficiently interact with complement C3 proteins, facilitating the hitchhiking of neutrophils through C3/C3R-mediated phagocytosis. As a result, LnNPs@PG achieves the dynamic monitoring of neutrophils during conditions such as cerebral ischemia/reperfusion and cutaneous wound healing. T cells play a crucial role in cancer immunotherapy, regulation and monitoring of T cell activity are essential for the prognostic assessment of cancer treatments that rely on immune activation. To this end, Zhou *et al.* presented a T-cell-targeting fusogenic liposomes (T-Fulips) to regulate and quantify T cell activity for cancer theranostics 39. The surface of T-Fulips containing two types of functional ligands, 2,2,6,6-tetramethylpiperidine (TEMP) groups that neutralizing ROS and anti-CD3 antibodies (aCD3) that targeting CD3 on T cells. In the bloodstream, the aCD3 efficiently guides T-Fulips to T cells, followed by fusing with T cells to allow the anchoring of TEMP onto T cells. Subsequently, the TEMP molecules effectively neutralize ROS, thereby protecting T cells from oxidation-induced loss of activity. Moreover, TEMP undergoes a conversion into paramagnetic 2,2,6,6-tetramethylpiperidine 1-oxyl (TEMPO) radicals, enabling the quantification of T cell activity using magnetic resonance imaging. The ability to precisely visualize and quantify cellular behaviors *in vivo* offers great potential for personalized medicine and tailored treatment approaches. As these novel strategies continue to evolve, they have the potential to significantly impact disease management, improve treatment outcomes, and advance the field of non-invasive diagnostic imaging. Continued research and clinical validation are crucial to further unlock the full potential of hitchhiking-based imaging probes and their applications in precision medicine.

## Summary and perspectives

Hitchhiking, which involves cruising on surfaces, has been adapted as a bio-inspired cargo delivery system for various applications. The rapid development of nanotechnology enables nanomaterials to interact with circulatory cells or proteins, either by phagocytosis or adsorption, thereby facilitating *in vivo* hitchhiking. In this system, circulatory cells (neutrophils, red blood cells, etc.) or proteins (serum albumin, ApoE, β2-GPI, Vtn, etc.) play a pivotal role as innate “moving gears” that facilitate the transport of hitchhiking nanoparticles to their desired target site. By combining the advantages of synthetic nanomaterials with circulatory cells/proteins, hitchhiking nanomaterials offer numerous benefits compared to conventional vectors. These advantages include enhanced circulatory stability, enhanced biosafety, and particularly improved targeting efficacy. The traditional ligand mediated active targeting is greatly influenced by the formation of protein corona in serum. However, by engineering the nanoparticle surface to preferentially adsorb certain types of proteins, hitchhiking strategies demonstrate an optimized protein corona that facilitates ligand-receptor interactions with specific target cells or tissues. This approach help overcome some of the limitations imposed by the protein corona and improve the nanoparticles' overall targeting efficacy. Consequently, hitchhiking nanomaterials have emerged as promising drug delivery vectors in various fields, such as anti-inflammation, cancer treatment, gene therapy, and diagnostic imaging.

Indeed, most hitchhiking systems involve the use of both cell-derived and synthetic materials, which present significant challenges in gaining regulatory approval for clinical applications. To ensure the safe and effective use of these systems, a series of standard and rigorous evaluations should be conducted prior to their implementation in clinical research. These evaluations should include the following: (1) Biocompatibility studies: thorough biocompatibility studies are essential to understand the interactions between hitchhiked cells and synthetic materials. These studies should assess potential adverse effects on cell function, viability, and immune response. Identifying any unexpected consequences of hitchhiking will enable researchers to optimize the system for safety and efficacy. (2) Immunogenicity assessment: The potential immunogenic response of the hitchhiking system should be carefully evaluated, especially when using foreign materials or nanoparticles. Understanding how the immune system responds to hitchhiked cells and materials is crucial for predicting possible immune reactions upon administration *in vivo*. (3) Long-term safety and toxicity: comprehensive investigations of the long-term safety and potential toxicity of the hitchhiking system are necessary. Researchers should assess how the attached materials degrade over time and whether they accumulate in the body, as such accumulation could lead to adverse effects. (4) *In vivo* studies: Conducting animal studies is crucial to evaluate the overall safety and efficacy of the hitchhiking system. These studies provide valuable insights into the distribution, biodistribution, and clearance of hitchhiked cells and materials in a living organism. By performing these evaluations meticulously, researchers may address regulatory concerns and pave the way for the safe and successful translation of hitchhiking systems from the laboratory to clinical applications. This comprehensive approach ensures that the potential benefits of hitchhiking in targeted drug delivery and imaging are harnessed while safeguarding patient safety and regulatory compliance.

Despite the significant success achieved by hitchhiking nanomaterials, there are still some challenges that limit their widespread application. To overcome these limitations, further efforts should be directed toward the following aspects: i) Targeting efficiency and specificity. Enhancing the targeting efficiency and specificity of nanomaterials to circulatory cells/proteins is crucial for their effective hitchhiking and delivery to specific tissues or cells. Further research is needed to optimize the selection and design of ligands or functional groups on the surface of nanomaterials to ensure efficient recognition and binding to the desired target. Ii) Precisely controlled drug release. The premature release of loaded drugs during hitchhiking may impair the migration, homing, and other functions of hitchhiked cells/proteins, resulting in unpredictable therapeutic efficacy. Further studies are needed to optimize the release behaviors of loaded drugs to ensure the precisely controlled release of the drug in the target. Iii) Scalability and manufacturing: To facilitate the practical application of hitchhiking nanomaterials, scalable and reproducible manufacturing methods need to be developed.

## Figures and Tables

**Table 1:** Advanced hitchhiking nanomaterials for biomedical applications.

Nanomaterials	Cells/Proteins	Hitchhiking way	Signaling pathway	Cargoes	Applications	Ref
Nano-integrated cascade enzymes	Neutrophils	Internalization	Fc*γ* receptor (Fc*γ*R) III	SOD/CAT	Ischemic stroke treatment	135
PLGA-PEG-based nanoparticle	Neutrophils	Internalization	CFLFLF peptide	Ligustrazine	Ischemic stroke treatment	52
Bacteria-derived outer-membrane vesicle	Neutrophils	Internalization	Fc*γ* receptor (Fc*γ*R) III	Pioglitazone	Ischemic stroke treatment	84
Denatured BSA nanoparticles	Neutrophils	Internalization	Fc*γ* receptor (Fc*γ*R) III	Doxorubicin	Ischemic stroke treatment	103
Denatured BSA nanoparticles	Neutrophils	Internalization	Fc*γ* receptor (Fc*γ*R) III	TPCA-1 (NF-*κ*B inhibitor)	Acute lung injury treatment	102
DOCP lipid nanoparticle	Neutrophils	Internalization	C3/C3R interaction	Dexamethasone/ampicillin	Acute lung injury treatment	57
HA-decorated multistage nano-micelles	Macrophages and neutrophils	Internalization	CD44/HA interaction	KIRA6 and Dexamethasone	Acute lung injury treatment	140
Lipid-conjugated floxuridine oligonucleotide	Serum albumin	Adsorption	Hydrophobic interaction	LFU20	Cancer treatment	123
Hitchhiking hybrid micelles (POR/DT)	Serum albumin	Adsorption	Hydrophobic interaction	OXA/TRE	Cancer treatment	144
OPDEA-PSN38	Red blood cells	Adsorption	Interaction with the PE and PC	SN38	Cancer treatment	82
N-oxide moiety-containing liposomes	Red blood cells	Adsorption	Interaction with the PE and PC	SN38	Cancer treatment	81
5-HT equipped nanoparticles	Neutrophils	Internalization	5-HT/MPO interaction	HPPH/Zileuton	Cancer treatment	145
Anti-CD11b BSA nanoparticle	Neutrophils	Internalization	Anti-CD11b/CD11b interaction	Decitabine/IR820	Cancer immunotherapy	146
Anti-CD3e f(ab')2-decorated nanoparticle	T cells	Internalization	CD3/anti-CD3 interaction	pDNA	Cancer immunotherapy	62
Liver SORT lipid	Apolipoprotein E (ApoE)	Adsorption	ApoE/LDL-R interaction	mRNA	Gene therapy	152,153
Spleen SORT lipid	β2-glycoprotein I (β2-GPI)	Adsorption	β2-GPI/Phosphatidyl serine	mRNA	Gene therapy	152,153
Lung SORT lipid	Vitronectin (Vtn)	Adsorption	Vtn/α_v_β_3_ integrin	mRNA	Gene therapy	152,153
O-series LNPs	Apolipoprotein E (ApoE)	Adsorption	ApoE/LDL-R interaction	mRNA	Gene therapy	154,155
N-series LNPs	Fibrinogen	Adsorption	-	Tsc3 mRNA	Gene therapy	154,155
DOPG lipid nanoparticle	Neutrophils	Internalization	C3/C3R interaction	NIR-IIb immunotracer	Diagnostic imaging	105
T-cell-targeting fusogenic liposomes	T cells	Internalization	Anti-CD3/CD3 interaction	TEMP	Diagnostic imaging	39
